# Возобновляющаяся гипогликемия и структура сосудистых сплетений боковых желудочков головного мозга крыс

**DOI:** 10.14341/probl13579

**Published:** 2026-01-18

**Authors:** О. А. Фоканова, Т. В. Кораблёва, Н. Б. Медведева, П. К. Телушкин

**Affiliations:** Ярославский государственный медицинский университетРоссия; Yaroslavl State Medical UniversityRussian Federation

**Keywords:** возобновляющаяся гипогликемия, сосудистые сплетения мозга, боковые желудочки, recurrent hypoglycemia, choroid plexus, lateral ventricles

## Abstract

**ОБОСНОВАНИЕ:**

ОБОСНОВАНИЕ. Возобновляющаяся гипогликемия возникает при инсулинотерапии пациентов с сахарным диабетом и является существенной причиной нарушений функций мозга у этих больных. Большое значение в обеспечении деятельности головного мозга имеет функционирование гематоликворного барьера и производство спинномозговой жидкости (СМЖ). Основной объем СМЖ образуют сосудистые сплетения боковых желудочков (БЖ) головного мозга и глимфатическая система мозга. Роль сосудистых сплетений в развитии нарушений функций мозга при гипогликемии недостаточно изучена.

**ЦЕЛЬ:**

ЦЕЛЬ. Цель настоящей работы — определение структуры сосудистых сплетений боковых желудочков головного мозга при возобновляющейся гипогликемии.

**МАТЕРИАЛЫ И МЕТОДЫ:**

МАТЕРИАЛЫ И МЕТОДЫ. Объектом исследования были крысы, перенесшие 9 гипогликемических состояний после введения инсулина (с интервалом 3 дня, уровень глюкозы в крови 1,4–1,8 ммоль/л) и интактные животные. Оценивали объем БЖ и объемные фракции сосудистых сплетений в БЖ: относительный объем и общую фракцию сосудов, фракции клеток и соединительной ткани. Также регистрировали морфологические изменения эпителия сосудистого сплетения и нервной ткани перивентрикулярных пространств.

**РЕЗУЛЬТАТЫ:**

РЕЗУЛЬТАТЫ. У животных, перенесших серию гипогликемий, увеличивается максимальная площадь сечения БЖ, относительный объем сосудистых сплетений БЖ и объемная фракция сосудов, приходящаяся на объем сосудистого сплетения БЖ. Объемная фракция клеток, приходящаяся на объем сосудистого сплетения БЖ у крыс с возобновляющейся гипогликемией, уменьшается. При морфологическом исследовании у этих животных регистрируются дистрофические изменения эпителиоцитов сосудистого сплетения и зоны дистрофических изменений в ткани головного мозга, окружающей желудочки.

**ЗАКЛЮЧЕНИЕ:**

ЗАКЛЮЧЕНИЕ. Таким образом, возобновляющаяся гипогликемия приводит к увеличению максимальной площади сечения БЖ и относительного объема сосудистых сплетений, а также дистрофическим изменениям клеток эпителия сосудистых сплетений и нейронов перивентрикулярных пространств. Поскольку гипогликемия наблюдается в ходе лечения пациентов с сахарным диабетом неоднократно, выявленные изменения могут стать причиной нарушения когнитивных функций и развития деменции у этих больных.

## Abstract (en)

**BACKGROUND:**

BACKGROUND. Recurrent hypoglycemia occurs during insulin therapy in patients with diabetes mellitus and is a significant cause of brain dysfunction in these patients. The functioning of the hematocerebrospinal fluid barrier and the production of cerebrospinal fluid (CSF) are of great importance in ensuring brain activity. The main volume of CSF is formed by the choroid plexus of the lateral ventricles (LV) of the brain and the glymphatic system of the brain. The role of the choroid plexus in the development of brain dysfunction in hypoglycemia has not been sufficiently studied.

**OBJECTIVE:**

OBJECTIVE. The aim of this work was to determine the structure of the choroid plexuses of the lateral ventricles of the brain during recurrent hypoglycemia.

**MATERIALS AND METHODS:**

MATERIALS AND METHODS. The object of the study were rats that had undergone 9 hypoglycemic states after insulin administration (with an interval of 3 days, the blood glucose level of 1.4–1.8 mmol/L) and intact animals. The volume of the LV and volume fractions of the choroid plexuses in the LV were estimated: the relative volume and total fraction of vessels, fractions of cells and connective tissue. Morphological changes in the epithelium of the choroid plexus and nervous tissue of the periventricular spaces were also recorded.

**RESULTS:**

RESULTS. In animals that had undergone a series of hypoglycemias, the maximum cross-sectional area of the LV, the relative volume of the choroid plexuses of the LV and the volume fraction of vessels per volume of the choroid plexus of the LV increase. The volume fraction of cells per volume of the choroid plexus of the ventricles decreases in rats with recurrent hypoglycemia. Morphological examination of these animals reveals dystrophic changes in the epithelial cells of the choroid plexus and zones of dystrophic changes in the brain tissue surrounding the ventricles.

**CONCLUSION:**

CONCLUSION. Thus, recurrent hypoglycemia leads to an increase in the maximum cross-sectional area of the LV and the relative volume of the choroid plexuses, as well as dystrophic changes in the epithelial cells of the choroid plexuses and neurons of the periventricular spaces. Since hypoglycemia is repeatedly observed during the treatment of patients with diabetes mellitus, the identified changes may cause cognitive impairment and the development of dementia in these patients.

## ОБОСНОВАНИЕ

Возобновляющаяся гипогликемия (ВГ) возникает при инсулинотерапии сахарного диабета и является существенной причиной нарушений функций мозга у пациентов с сахарным диабетом (СД) 1-го и 2-го типов. При этом повторные эпизоды гипогликемии вызывают снижение контррегуляторного и вегетативного ответов, что ведет к нарушению распознавания пациентом гипогликемии и увеличивает риск развития последующих гипогликемий [1–5].

Мозг исключительно зависим от снабжения глюкозой. Гипогликемия приводит к нарушению функций нейронов, и в основе повреждения клеток мозга лежит глутаматная эксайтотоксичность [5–7].

Большое значение в обеспечении работы головного мозга имеет функционирование гематоликворного барьера и производство спинномозговой жидкости (СМЖ, ликвор). Основной объем СМЖ образуют сосудистые сплетения боковых желудочков (БЖ) головного мозга и глимфатическая система мозга [8][9]. Эпителиальные клетки сосудистых сплетений секретируют в ликвор множество метаболитов, нейротрофических и ангиогенных факторов и участвуют в процессах восстановления нейронов после травмы или инсульта. Повреждение сосудистых сплетений наблюдаются при различных патологических состояниях — менингите, ишемическом инсульте, гипоксии и при первичных нейродегенеративных расстройствах [9]. Роль сосудистых сплетений в развитии нарушений функций мозга при гипогликемии недостаточно изучена.

## ЦЕЛЬ

Цель настоящей работы — определение структуры сосудистых сплетений боковых желудочков головного мозга крыс при возобновляющейся гипогликемии.

## МАТЕРИАЛЫ И МЕТОДЫ

Объектом исследования были 15 белых крыс линии Вистар массой 200–220 г, разделенных на 2 группы: 1-я группа — контроль (интактные животные) — 5 крыс, 2-я группа — животные, перенесшие серию гипогликемических состояний (ВГ) — 15 крыс.

Содержание животных соответствовало правилам лабораторной практики при проведении доклинических исследований в РФ (ГОСТ 351000.3-96 и 51000.4-96) и Приказу МЗ РФ №267 от 19.06.2003 г. «Об утверждении правил лабораторной практики» с соблюдением Международных рекомендаций Европейской конвенции по защите позвоночных животных. Все эксперименты проведены в соответствии с отечественными нормативами и современными международными биоэтическими стандартами по работе с лабораторными животными; на эксперименты получено заключение локального этического комитета ЯГМУ, протокол №4 от 18.10.2016.

Перед опытом крысы были лишены пищи в течение 14–16 часов. Во все время эксперимента животные находились в состоянии свободного доступа к воде. Гипогликемические состояния вызывали внутримышечным введением инсулина (Actrapid MC, 40 ЕД/кг массы тела). Через 2–3 часа после инъекции инсулина у крыс наблюдалась связанная с гипогликемией неврологическая симптоматика: потеря постуральных рефлексов — достижение бокового положения без попыток поднять голову. При этом на протяжении эксперимента в каждый день опыта у пяти отдельных животных в состоянии гипогликемии определяли уровень глюкозы в крови из хвостовой вены, полученной после удаления небольшого участка хвоста, глюкозоксидазным методом (глюкоза-АГАТ). В условиях эксперимента уровень глюкозы в крови у крыс с гипогликемией составлял 1,4–1,8 ммоль/л.

Сразу после утраты постуральных рефлексов гипогликемическое состояние купировали введением 5 мл 40% раствора глюкозы в желудок через зонд. Эксперимент повторяли каждые 3 суток. Всего животные перенесли 9 гипогликемических состояний.

Крысы выводились из эксперимента во время последней гипогликемии путем декапитации. Головной мозг извлекали и переносили в фиксатор — 10% нейтральный формалин и заливали в гистомикс. Горизонтальные серийные срезы изготавливали на роторном микротоме (pfm Rotary 3003 — Rotation Microtome) толщиной 4–5 мкм, окрашивали гематоксилином и эозином с последующим заключением в бальзам. Для анализа использовали не менее 50 срезов, в которых находились боковые желудочки (БЖ) головного мозга от каждого животного. Микрофотографии получали при помощи цифрового микроскопа со встроенной фотокамерой Motic DM-1802-A. Для оценки объема БЖ выбирали срезы (×4), где площадь сечения желудочков была максимальной. Показатель определяли путем обведения границ БЖ и последующих расчетов в морфометрической компьютерной программе ImageJ. Определение объемных фракций сосудистых сплетений в БЖ: относительного объема и общей фракции сосудов, фракции клеток и соединительной ткани проводили стереологически на 50 серийных срезах с помощью окулярной сетки с 60 равноудаленными узлами пересечения на микроскопе Микромед при увеличении в 400 раз.

При статистической обработке для проверки однородности дисперсий полученных данных использовали критерий Фишера. В сравниваемых выборках условие гомоскедастичности выполнено. Распределение значения переменных в вариационных рядах первичных данных оценивали с помощью критерия Колмогорова-Смирнова. Распределение переменных было нормальным, поэтому проверку статистических гипотез проводили с помощью параметрических методов t-критерия Стьюдента. Данные представлены как среднеарифметическое значение и стандартная ошибка средней (М±m). Различия считали статистически значимыми при уровне значимости p<0,05.

## РЕЗУЛЬТАТЫ

У животных, перенесших серию гипогликемий, максимальная площадь сечения БЖ увеличивается на 23%, относительный объем сосудистых сплетений БЖ и объемная фракция сосудов в сплетениях увеличиваются соответственно на 14% и 18%, а объемная фракция клеток в сплетениях уменьшается на 15% (во всех случаях p<0,05) (табл. 1).

**Table:** Таблица 1. Изменения количественных показателей структуры боковых желудочков головного мозга крыс при возобновляющейся гипогликемии (ВГ) (М±m) Примечание: n — количество животных в группе, * — p<0,05.

Группа	Максимальная площадь сечения в расчете на один желудочек, мм²	Относительный объем сосудистых сплетений, %	Объемная фракция сосудов в сплетениях, %	Объемная фракция соединительной ткани в сплетениях, %	Объемная фракция клеток в сплетениях, %
Контроль (n=5)	2,2±0,1	65±3	39±2	9,0±0,6	52±3
ВГ (n=15)	2,7±0,2*	74±2*	46±2*	10,0±1,0	44±1*

Таким образом, в БЖ мозга крыс при возобновляющейся гипогликемии зарегистрированы серьезные морфофункциональные преобразования: происходит увеличение объема желудочков, в желудочках наблюдается увеличение относительного объема сосудистых сплетений, в которых повышается объемная фракция сосудов и снижается объемная фракция клеток (табл. 1).

В сосудистых сплетениях наблюдаются дистрофические изменения эпителиоцитов: клетки увеличены в объеме, ядро смещенно на периферию, обнаруживается гомогенизация и инкрустрация цитоплазмы этих клеток. При этом в разных участках сосудистых сплетений видны спазмированные и переполненные капилляры, а также участки отслоения эпендимы от подлежащей ткани мозга и десквамация клеток эпителия сосудистого сплетения (рис. 1). Такие нарушения способны приводить к некрозу клеток, следствием чего может быть снижение объемной фракции клеток в сплетениях (табл. 1).

**Figure:**
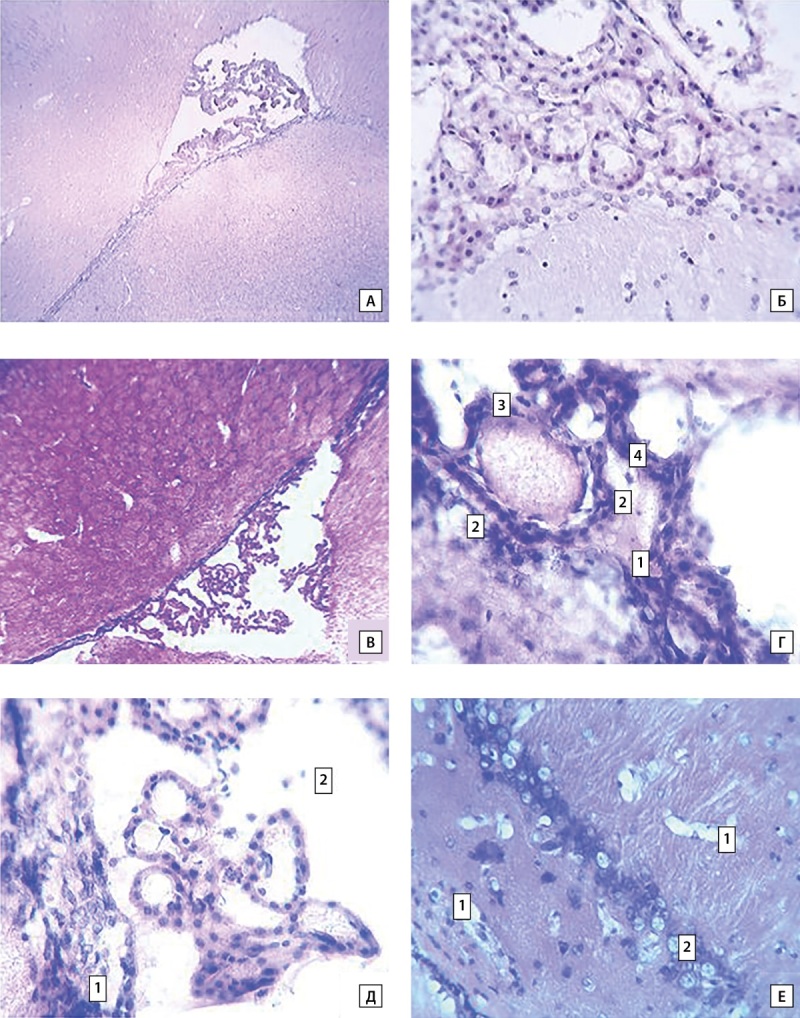
Рисунок 1. Структура боковых желудочков и перивентрикулярной ткани головного мозга крыс при возобновляющейся гипогликемии. Обозначения: А. Боковой желудочек (общий вид) — контроль. Ув.:40; Б. Сосудистые сплетения — контроль. Ув.:400; В. Боковой желудочек (общий вид) в эксперименте. Ув.:40; Г. Сосудистые сплетения в эксперименте: 1 — выпадение нитей фибрина, 2 — набухшие эпителиоциты, 3 — перерастянутый капилляр, 4 — спазмированный гемокапилляр. Ув.:400; Д. Сосудистые сплетения в эксперименте: 1 — отслоение эпендимы от подлежащей ткани мозга, 2 — экструзия клеток эпителия сосудистого сплетения. Ув.:400; Е. Перивентрикулярная ткань головного мозга в эксперименте: 1 — периваскулярный отек вен, 2 — перицилюлярный отек нейронов. Ув.:400.

Зоны дистрофических изменений клеток и выраженные перицеллюлярные и периваскулярные отеки регистрируются и в ткани головного мозга, окружающей желудочки (рис. 1). При этом более повреждены клетки, находящиеся вблизи цереброспинальной жидкости. Также по периферии желудочков головного мозга визуально наблюдается увеличение числа глиальных клеток.

## ОБСУЖДЕНИЕ

У животных, перенесших серию гипогликемических состояний, наблюдается увеличение максимальной площади сечения БЖ и относительного объема сосудистых сплетений (табл. 1).

Увеличение сосудистых сплетений обнаруживается при множестве заболеваний, связанных с нарушением функций мозга, таких как менингит, депрессия, психоз, шизофрения, комплексный регионарный болевой синдром, эпилепсия, ишемический инсульт, гипоксия, ожирение, а также при первичных нейродегенеративных расстройствах, таких как болезнь Альцгеймера, лобно-височная деменция, болезнь Хантингтона, болезнь Паркинсона и нормальное старение [9]. Таким образом, увеличение объема сосудистых сплетений не является специфичным для возобновляющейся гипогликемии, но служит свидетельством развития патологического процесса.

Поскольку объемная фракция клеток в сплетениях снижается, а объемная фракция соединительной ткани в сплетениях не изменяется (табл. 1.), то увеличение относительного объема сосудистых сплетений обусловлено, по-видимому, повышением объемной фракции сосудов.> Такие изменения могут иметь компенсаторный характер и быть направлены на увеличение продукции СМЖ [10]. Снижение способности к продукции ликвора у животных с возобновляющейся гипогликемией представляется вероятным, поскольку количество клеток сосудистого сплетения уменьшается, и эпителиальные клетки обнаруживают явные дистрофические изменения (табл. 1, рис. 1).

Основным механизмом повреждения нейронов при гипогликемии, гипоксии/ишемии и нейродегенеративных заболеваниях является эксайтотоксичность, связанная с значительным увеличением уровня глутамата в межклеточных пространствах и избыточной активацией нейрональных N-метил-D-аспартат (NMDA) рецепторов глутамата [5–7]. При гипогликемии в межклеточной среде, кроме увеличения уровня глутамата, значительно увеличивается количество аспартата [11], также лиганда NMDA рецепторов [12].

Гиперактивация NMDA-рецепторов, которые представляют собой Na⁺-Са²⁺ лиганд-зависимые ионные каналы, приводит к увеличению входа Са²⁺ в нейроны и в митохондрии. Увеличение концентрации Са²⁺ в митохондриях вызывает открытие пор митохондриальной проницаемости (mPTP) и падение тем самым трансмембранного потенциала внутренней мембраны митохондрий. Это сопровождается увеличением продукции активных форм кислорода (ROS) — супероксиданионрадикала (O2•), перекиси водорода (H2O2), которая легко проходит через клеточные мембраны, и гидроксильного радикала (•OH). Накопление ROS приводит к повреждению ядерной ДНК и активации поли(АДФ-рибоза) полимеразы-1, стимулирует проапоптотические сигнальные пути и вызывает клеточное старение. Постоянное открытие mPTP сопровождается изменениями осмотического давления и набуханием митохондрий, что в итоге приводит к их лизису и гибели, регулируя некроз клеток [5–7][13].

Удаление потенциально опасных метаболитов из межклеточных пространств мозга осуществляется глимфатической системой, которая представляет собой организованный поток СМЖ через периваскулярные пространства на уровне паренхиматозных капилляров мозга и внутри паренхиматозного интерстиция мозга и регулируется каналами аквапорина 4 [9]. В результате глимфатического потока осуществляется значительная часть продукции и абсорбции спинномозговой жидкости [8][14]. Посредством глимфатической системы вещества из межклеточных пространств мозга попадают в желудочковую СМЖ. Таким образом аспартат, глутамат и перекись водорода, которые выделяются в межклеточные пространства при гипогликемии, поступают в СМЖ подобно тому, как это происходит при ишемии, когда уровень глутамата в ликворе увеличивается в 55 раз [15].

Эпителиоциты сосудистого сплетения и эпендимальные клетки желудочков экспрессируют NMDA рецепторы, которые сосредоточены в люменальной мембране [15].> Поэтому> неоднократно возникающие при возобновляющейся гипогликемии увеличения концентрации аспартата и глутамата в СМЖ, приводя к активации NMDA-рецепторов и массивному поступлению Ca²⁺ в эпителиальные клетки сосудистых сплетений, способны вызывать их эксайтотоксическое повреждение. С этим, по-видимому, связаны повреждения эпителия сосудистого сплетения и уменьшение количества клеток сплетения (табл. 1, рис.1), выявленные в настоящем исследовании у крыс с возобновляющейся гипогликемией.> Кроме того, при глутаматной эксайтотоксичности увеличение продукции активных форм кислорода повышает экспрессию и выделение клетками сосудистых сплетений матриксной металлопротеиназы 9 — внеклеточной цинк-зависимой эндопептидазы, способной разрушать все типы белков внеклеточного матрикса, включая белки плотных контактов и базальные ламинарные белки, что может приводить к нарушению гематоликворного барьера [13].

Функцией сосудистого сплетения является и активная защита головного и спинного мозга путем секреции множества нейротрофических и ангиогенных факторов из эпителиальных клеток в спинномозговую жидкость. К этим факторам относятся инсулиноподобный фактор роста 2 (IGF2), трансформирующий фактор роста-b (TGF-b), транстиретин и многие другие, включая инсулин [9][16]. Таким образом, выявленные в настоящем исследовании дистрофические повреждения эпителия сосудистого сплетения могут иметь неблагоприятные последствия для мозга в целом.

Одной из причин, выраженных перицеллюлярных и периваскулярных отеков и дистрофических изменений клеток в ткани головного мозга, окружающей желудочки (рис. 1) может быть эксайтотоксичность. Эпендимоциты желудочков мозга экспрессируют высокоэффективные переносчики глутамата, которые, вероятно, отвечают за поддержание низких концентраций глутамата в ликворе. При этом глутамат поступает в перивентрикулярную паренхиму мозга, и уровень его в нервной ткани, окружающей желудочки, может значительно превышать концентрацию в СМЖ [15]. Переходу глутамата, аспартата и активных форм кислорода в перивентрикулярные пространства способствует отсутствие плотных соединений между эпендимальными клетками [17] и ток жидкости в глимфатической системе в пространствах между волокнистыми путями в белом веществе, прилежащем к субэпендимальной паренхиме [18].

Преимущественное поражение перивентрикулярных нейронов и нейронов поверхностных слоев коры больших полушарий головного мозга наблюдается при тяжелой продолжительной гипогликемии (уровень глюкозы в крови менее 1,0 ммоль/л в течение 10–30 мин), сопровождающейся развитием «изоэлектрической» ЭЭГ [19]. Однако степень гипогликемии, индуцированной в этих исследованиях, является экстремальной и редко наблюдается у людей с сахарным диабетом [5]. В нашем эксперименте уровень глюкозы в крови животных при гипогликемии находился в пределах 1,4–1,8 ммоль/л и гипогликемическое состояние немедленно купировалось введением глюкозы. Таким образом, повторяющиеся эпизоды относительно умеренной кратковременной гипогликемии и долгосрочная тяжелая (претерминальная) гипергликемия приводят к сходным нарушениям в мозге. Кроме того, повреждение нейронов при ВГ может быть потенцировано нарушением синтеза различных факторов роста в эпителиальных клетках сосудистого сплетения, повреждение которых выявлено в настоящем исследовании.

## ЗАКЛЮЧЕНИЕ

Таким образом возобновляющаяся гипогликемия приводит к увеличению максимальной площади сечения БЖ и относительного объема сосудистых сплетений, а также дистрофическим изменениям эпителия сосудистых сплетений и нервной ткани перивентрикулярных пространств. Поскольку гипогликемия наблюдается в ходе лечения пациентов с сахарным диабетом неоднократно, выявленные изменения могут явиться одной из причин нарушения когнитивных функций и развития деменции у этих больных.

## ДОПОЛНИТЕЛЬНАЯ ИНФОРМАЦИЯ

Источники финансирования. Плановая НИР ФГБОУ ВО ЯГМУ Минздрава России.

Конфликт интересов. Авторы декларируют отсутствие явных> и потенциальных конфликтов интересов, связанных с содержанием> настоящей статьи.

Участие авторов. Фоканова О.А., Кораблёва Т.В., Медведева Н.Б. — отработка методики исследований, сбор и статистическая обработка материала, анализ и интерпретация данных, написание и редактирование текста; Телушкин П.К. — концепция и дизайн исследования, анализ и интерпретация данных, написание и редактирование текста. Все авторы внесли значимый вклад в проведение исследования и подготовку статьи, прочли и одобрили финальную версию статьи перед публикацией.
